# Coronene Bisimide as a Visible‐Light‐Mediated Triplet‐Triplet Energy Transfer Catalyst

**DOI:** 10.1002/chem.202502602

**Published:** 2025-09-08

**Authors:** Divya P. Sukumaran, Frank Würthner

**Affiliations:** ^1^ Julius‐Maximilians‐Universität Würzburg Institut für Organische Chemie Würzburg 97074 Germany; ^2^ Center for Nanosystems Chemistry (CNC) Julius‐Maximilians‐Universität Würzburg Würzburg 97074 Germany

**Keywords:** aromatic bisimides, catalysis, energy transfer, intersystem crossing, photosensitizer

## Abstract

Photosensitization has emerged as a versatile tool to facilitate access to excited states under mild conditions, allowing for efficient and selective photochemical transformations. Herein, we report a very simple molecule, coronene bisimide (CBI), as a potent visible‐light photosensitizer featuring a high extinction coefficient with a broadband absorption spanning from ultraviolet to green region of the visible spectrum, along with a long‐lived triplet state generated via efficient intersystem crossing (ISC). Utilizing the triplet‐triplet energy transfer (TTEnT) strategy, CBI catalyzes diverse reactions under green light irradiation. Through this, we propose CBIs as a new class of heavy‐atom‐free visible‐light sensitizers overcoming the limitations of conventional sensitizers, such as low extinction coefficient in the visible regime, short triplet‐state lifetimes, and high toxicity, by relying on heavy atoms.

## Graphical abstract

Coronene bisimide (CBI) with its efficient intersystem crossing was used as a visible‐light‐absorbing photosensitizer. Different reactions, including cycloadditions and oxidations, were demonstrated via triplet‐energy transfer from CBI.

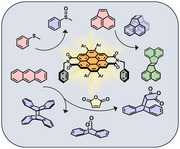

Photoexcitation expands the reactivity of organic molecules by accessing excited states of unique reactivity profiles on the higher excited‐states potential surfaces. In particular, accessing triplet states diversifies the reaction pathways, enabling radical‐like transformations and making use of extended excited state lifetimes. Direct excitation of organic reactants is often unsustainable, as they usually require high‐energy UV photons, resulting in the lack of selectivity and efficiency with typically slow intersystem crossing (ISC) rates. Thus, triplet‐triplet energy transfer (TTEnT) from photosensitizers to the desirable substrates evolved as an efficient strategy to circumvent these issues, as it delivers milder reaction conditions and offers enhanced selectivity.^[^ ^1^ ^]^ An ideal photocatalyst for TTEnT‐ sensitization should show a high absorption cross‐section at the desired wavelength of excitation, afford efficient ISC to its triplet state, provide long triplet state lifetime, and also higher triplet excited‐state energy compared to the targeted substrate.^[^ ^2^ ^]^ Heavy‐atom effect, introduced by incorporating atoms of large atomic number to induce strong spin–orbit coupling, promotes efficient ISC and accordingly became an established approach for unlocking these triplet states.^[^ ^3^ ^]^ Nevertheless, the incorporation of heavy atoms suffers from drawbacks such as short triplet‐state lifetimes in conjunction with increased cost of preparation and high toxicity.^[^ ^4^ ^]^ As a promising alternative to such heavy‐atom‐containing traditional photocatalysts, heavy‐atom‐free donor–acceptor‐based thermally activated delayed fluorescence (TADF) photocatalysts have emerged in recent years with notable potential in photocatalysis. However, as this category of catalysts relies on charge‐transfer transitions, they possess low oscillator strength in the visible range, and the catalytic potential is strongly influenced by the solvent environment, as the energy of charge‐transfer states is solvent polarity‐dependent.^[^ ^5^ ^]^

From the photophysical perspective, the bis(dicarboximides) of polycyclic aromatic hydrocarbons (PAHs),^[^ ^6^ ^]^ such as naphthalene and perylene bis(dicarboximides) (NBIs, PBIs) (Figure 1a), are intriguing dyes, along with their electronic properties and high photochemical as well as thermal stability, which have made them also attractive in the field of photoredox catalysis over the last decade.^[^ ^7^, ^8^ ^]^ The inherently limited absorptivity of NBIs in the visible spectral regime, without any core substitution,^[^ ^9^ ^]^ represents the foremost challenge in developing visible‐light‐driven photocatalysts featuring NBI core. On the other hand, PBIs, with broad visible‐light absorption and high molar extinction coefficient in combination with tunable optoelectronic features by simple structural modifications,^[^ ^10^ ^]^ are superior candidates which have been introduced in their photoreduced state as low‐cost alternatives for metal‐based photoredox catalysts.^[^ ^11^, ^12^, ^13^ ^]^ In contrast, leveraging the outstanding optical properties of the neutral PBI molecules for TTEnT catalysis remains elusive because of an inefficient S_1_→T_1_ ISC in the absence of heavy atom substituents. ^[^ ^14^, ^15^ ^]^ More importantly, even if triplet states are populated in PBIs by heavy atoms, their energy is too low for the desired sensitization of substrates by TTEnT (Figure 1a). In this regard, coronene bisimide (CBI), obtained by simple twofold bay annulation of PBI, is promising owing to its favorable photophysical properties, especially the reduced S_1 _− T_1_ energy gap^[^ ^16^ ^]^ that leads to an enhanced ISC, enabling the access of an energetically high‐lying triplet state. Surprisingly, CBIs still appear to be unexplored in photocatalytic applications.

**Figure 1:**
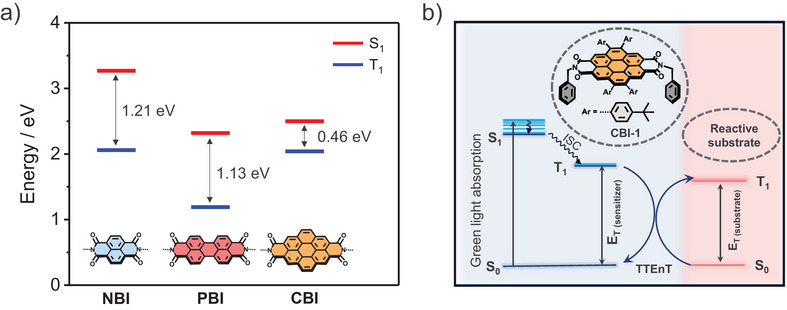
a) Energy level diagram comparing NBI, PBI, and CBI from the experimental data available in the literature. ^[^ ^17^, ^18^, ^19^ ^]^ b) Schematic representation of TTEnT from the sensitizer to the preferred substrate and the structure of **CBI**‐**1**.

In a previous study on CBI cyclophanes^[^ ^16^ ^]^ we noticed for the tetra(*tert*‐butylphenyl)‐substituted reference compound **CBI**‐**1** an efficient ISC in the solution state without any external heavy‐atom perturbation. **CBI**‐**1** shows a strong UV‐vis absorption spectrum with maxima at 529, 433, and 339 nm along with vibronic features and a slightly red‐shifted emission with a maximum at 548 nm for fluorescence, and a further red‐shifted phosphorescence at a maximum of 626 nm (Figure 2). The fluorescence quantum yield (QY) of **CBI**‐**1** is 48% with a lifetime of 5.34 ns and with a delayed component of 4.5 ms with QY of 3% when measured under inert conditions (Figure 2‐inset). Thus, this TADF process and very weak phosphorescence at room temperature in solution establish the efficient ISC taking place in **CBI**‐**1**. The triplet quantum yield was estimated to be 39% by employing TTEnT from **CBI**‐**1** to β‐carotene as the triplet acceptor, with [Ru(bpy)_3_]^2+^ as the standard.^[^ ^16^ ^]^ The broad absorption band spanning into the green range of the electromagnetic spectrum and a large extinction coefficient in this spectral range, along with enhanced ISC and the long triplet state lifetime of **CBI**‐**1,** are the evident attributes of a desirable visible‐light‐mediated photosensitizer.^[^ ^2^ ^]^

**Figure 2:**
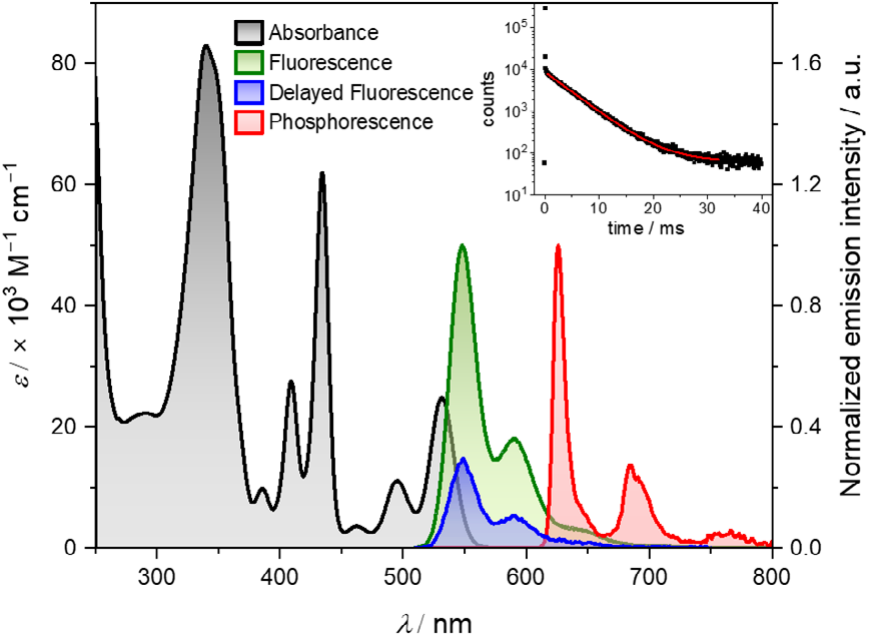
UV‐vis absorption spectrum (black), normalized emission spectrum (green), and delayed fluorescence spectrum (blue, normalized and scaled to ten times the quantum yield, gate delay: 0.05 ms, gate width: 10 ms, under N_2_) in CHCl_3_ at 293 K, as well as the normalized phosphorescence spectrum (red) in CHCl_3_ with 20% ethyliodide at 77 K of **CBI‐1**. The inset shows the fluorescence decay of **CBI‐1** in CHCl_3_ under N_2_ at 293 K.

Given these promising photophysical properties of **CBI**‐**1**, we explored the viability of this molecule as a triplet photosensitizer by conducting proof‐of‐concept reactions to illustrate the potential of **CBI‐1** (E_T _= 2.02 eV) in catalyzing a broad variety of reactions, which can be classified as cycloadditions and oxidations.

The reactions (vide infra) were performed by employing approximately 1–2 mol% of the catalyst and by utilizing low‐energy photons for excitation compared to the direct excitation of the substrate. All reactions were carried out in deuterated chloroform, considering the solubility of the substrates (for solubility of **CBI‐1** in some common solvents, see Table S1) and allowing monitoring of the reactions by ^1^H NMR spectroscopy. A home‐built setup with an LED of peak wavelength 520 nm (details in Supporting Information, Figure S1) was used for the irradiation of the samples. Reaction progress was monitored at different irradiation times and followed until completion. Additional experimental details and data (Figures S3–S13) are provided in the Supporting Information. Notably, no side products were detected, and the catalyst demonstrated excellent stability, which is confirmed from the spectra and other reference experiments (Figures S14,S15).

Energy transfer from sensitizer to substrate promoting triplet population is known to enable pericyclic transformations that are forbidden or inaccessible under thermal conditions. Accordingly, **CBI‐1** was used for [2 + 2], [4 + 2], and [4 + 4] cycloadditions of different substrates under green light excitation, which otherwise demands high‐energy photons or thermally harsh conditions. In a first study, the photodimerization of acenaphthylene was considered for a prototypical [2 + 2] transformation, which has already been extensively investigated with regard to the effect of sensitization, solvents, and the difference in the ratio of the *Z*/*E* isomers formed.^[^ ^20^, ^21^ ^]^ In our study, **CBI‐1** undergoes efficient ISC upon photoexcitation, and the triplet energy is transferred via the Dexter mechanism to acenaphthylene, which has a lower triplet energy (E_T _= 1.9–2.0 eV^[^ ^22^ ^]^). Thus, the dimerization of acenaphthylene with **CBI**‐**1** as a catalyst results in the formation of dimers with an anti:syn isomeric ratio of 1:4 under the given irradiation conditions (Figure 3a).

**Figure 3:**
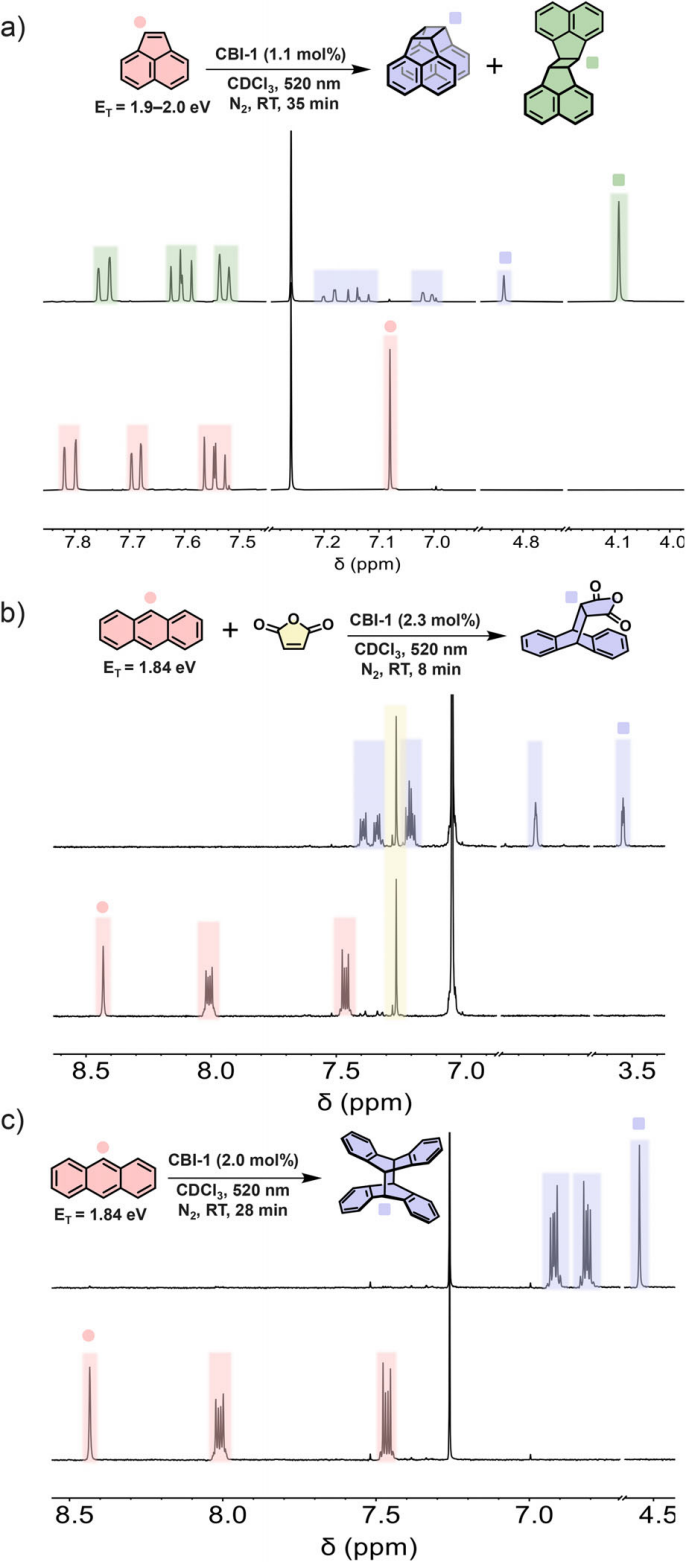
Partial ^1^H NMR spectra (400 MHz, CDCl_3_, 295 K, N_2_) showing a) acenaphthylene with **CBI**‐**1** ([acenaphthylene] = 2.6 × 10^−3^ M, [**CBI**‐**1**] = 3.0 × 10^−5^ M) before (bottom spectrum) and after (top spectrum) 35 minutes of irradiation; b) anthracene and maleic anhydride with **CBI**‐**1** ([anthracene] = 1.2 × 10^−3^ M, [maleic anhydride] = 4.0 × 10^−2^ M, [**CBI**‐**1**] = 2.8 × 10^−5^ M) before (bottom spectrum) and after (top spectrum) 8 minutes of irradiation; c) anthracene with **CBI**‐**1** ([anthracene] = 1.5 × 10^−3^ M, [**CBI**‐**1**] = 3.0 × 10^−5^ M) before (bottom spectrum) and after (top spectrum) 28 minutes of irradiation.

Subsequently, the Diels‐Alder reaction has been considered as an example of a thermally allowed [4 + 2] cycloaddition in agreement with the Woodward‐Hoffman rule. Even though the Diels‐Alder reaction is a powerful strategy in the formation of six‐membered rings, it has the pitfall of requiring harsh conditions like elevated temperatures and acidic environments for an enhanced reaction rate.^[^ ^23^, ^24^ ^]^ But leveling up the reaction pathway by photoexcitation accelerates the chemical transformations to rates unattainable in the ground state. Thus, with the same strategy of photosensitizing the reacting species, that is, via exciting **CBI**‐**1** followed by a TTEnT to anthracene with its low‐lying triplet state (E_T _= 1.84 eV ^[^ ^25^ ^]^), the Diels‐Alder‐like [4 + 2] cycloaddition at ambient temperature is enabled. The cycloaddition reaction of anthracene with maleic anhydride is reported here as a popular example of the sort (Figure 3b). While conducting the [4 + 2] cycloaddition of anthracene and maleic anhydride under the given catalytic conditions, we observed the competitive formation of anthracene dimer as the side product for higher concentrations of anthracene. This observation, attributed to the efficient [4 + 4] dimerization of anthracene in its excited triplet state, is one of the oldest known photoreactions that takes place via triplet‐triplet annihilation (TTA) of excited anthracene monomers.^[^ ^26^ ^]^ Thus, following TTEnT from **CBI**‐**1** to anthracene, two triplet excited anthracene molecules collide, generating singlet excited anthracene, which on further collision with ground state anthracene form the dimer, notably under green light irradiation. The NMR spectra monitoring the formation of the dimer from anthracene are shown in Figure 3c.

To broaden the scope of reactions beyond cycloadditions, **CBI**‐**1** was employed for oxidative transformations using singlet oxygen generated from ground‐state triplet oxygen by visible‐light‐mediated sensitization. In these reactions a sensitizer molecule with sufficient triplet energy (E_T _≥ 0.97 eV) can absorb visible light and transfer its energy to the ground‐state triplet oxygen species. The generated singlet oxygen is highly reactive and aids the transformation of the substrate of interest, which can be employed in natural product synthesis and biomedical applications like photodynamic therapy.^[^ ^27^ ^]^ We demonstrate here the oxidation of thioanisole and anthracene as two examples utilizing the singlet oxygen generation ability of **CBI**‐**1**. Albeit being of industrial interest as important synthons, the efficient and scalable synthesis of sulfoxides is challenging, as conventional oxidations often lead to overoxidation, resulting in toxic sulfones.^[^ ^28^ ^]^ The photocatalytic oxidation of thioanisole is chosen as a prototypical oxidation of sulfide and carried out employing **CBI**‐**1** as a catalyst. The electrophilic addition of singlet oxygen, followed by the addition of another sulfide molecule, results in the formation of sulfoxide ^[^ ^29^ ^]^ (Figure 4a), which, upon overoxidation on prolonged irradiation, can then be transformed into the corresponding sulfone under the same reaction conditions (Figure S9). Thus, irradiation time can be adjusted to control the formation of desirable products using **CBI**‐**1**.

**Figure 4:**
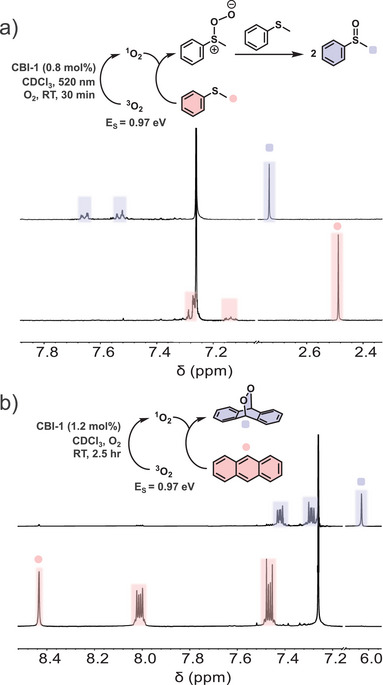
Partial ^1^H NMR spectra (400 MHz, CDCl_3_, 295 K) showing a) thioanisole with **CBI**‐**1** ([thioanisole] = 3.9 × 10^−3^ M, [**CBI**‐**1**] = 3.4 × 10^−5^ M) before (bottom spectrum) and after (top spectrum) 20 minutes of irradiation of an O_2_ saturated solution; b) anthracene with **CBI**‐**1** ([anthracene] = 2.6 × 10^−3^ M, [**CBI**‐**1**] = 3.3 × 10^−5^ M) before (bottom spectrum) and after (top spectrum) exposure to ambient light for 150 minutes.

As discussed earlier, electron‐rich anthracene can undergo [4 + 4] and [4 + 2] cycloadditions under inert conditions (Figure 3b,c). However, in the presence of oxygen, a [4 + 2] cycloadduct of anthracene and oxygen is formed even under ambient light conditions (Figure 4b). Herein, sensitization by **CBI**‐**1** results in the generation of singlet oxygen, which in a [4 + 2] cycloaddition affords anthracene endoperoxide. In contrast, irradiation with the stronger 520 nm LED light source generates a mixture of over‐oxidized anthracene derivatives (Figure S12).

To illustrate the involvement of the triplet state of **CBI**‐**1** in the above transformations, reactions in the presence of triplet quenchers were conducted, taking anthracene dimerization as the model reaction. In the presence of excess perylene (E_T _= 1.53 eV^[^ ^30^ ^]^) or diphenylanthracene (E_T _= 1.79 eV^[^ ^31^ ^]^) with lower‐lying triplet levels compared to anthracene (E_T _= 1.84 eV^[^ ^25^ ^]^), no dimerization of anthracene was observed (Figures S16–18). The presence of either perylene or diphenylanthracene can result in the TTEnT from anthracene to these quenching substrates for accessing their triplet states (Figure S19), thus preventing the diffusion‐controlled collision of two triplet‐excited anthracene molecules in the solution, which is a prerequisite for dimer formation.

Beyond the excellent absorption characteristics for visible light excitation, we consider the electron‐poor character of CBI as highly advantageous for a photosensitizer, similar to color pigments where many exhibit imide functionalities.^[^ ^32^ ^]^ To illustrate this point, the stability of **CBI‐1** has been established using anthracene dimerization as a representative example in the presence of an internal standard, showing the integrity and concentration of the catalyst remain constant after the reaction (Figures S14,15). A reaction on a larger scale was also carried out, providing a high yield of product. Accordingly, **CBI‐1** photocatalyst might be recycled easily for another reaction after separation using column chromatography (detailed in Supporting Information).

In summary, we presented **CBI**‐**1** as a versatile, purely organic, heavy‐atom‐free photosensitizer that can be employed to realize various photochemical transformations employing TTEnT. The distinct photophysical properties, including large absorption cross‐section, efficient ISC, and long‐lived triplet state, make **CBI‐1** a viable photosensitizer. The demonstrated reactions highlight the distinct advantage of **CBI**‐**1,** including low catalyst loading requirement, absence of side products, catalyst recyclability, along with the tunability of the reaction depending on the reaction conditions. Thus, CBIs with their long excited‐state lifetime and high (photo‐)stability provide an excellent addition to the library of visible‐light‐mediated triplet photosensitizers (Table S3). Further, considering the much better predictability of photofunctional properties in terms of structure‐property relationships for heavy‐atom‐free PAH imides compared to metal‐based sensitizers, we envision a plethora of new aromatic imides to be introduced as photocatalysts in the following years, inspired by the **CBI**‐**1** prototype.

## Supporting Information

Supporting information contains experimental details, reference experiment data, additional NMR spectra, turn‐over calculations, and summary table of photophysical properties of different available sensitizers. The authors have cited additional references within the Supporting Information. ^[^ ^2^, ^16^, ^33^, ^34^, ^35^, ^36^, ^37^, ^38^, ^39^, ^40^ ^]^

## Conflict of Interest

The authors declare no conflict of interest.

## Supporting information



Supporting Information

## Data Availability

The data underlying this study are openly available in the Supporting Information and in Zenodo at https://doi.org/10.5281/zenodo.16902617.
