# Supplementary material for: Coronene Bisimide as a Visible‐Light‐Mediated Triplet‐Triplet Energy Transfer Catalyst
Source: Chemistry. 2025 Sep 8;31(63):e02602. doi: 10.1002/chem.202502602 (PMC12619027; doi:10.1002/chem.202502602)
Supplement: Supplementary file 1 — Supporting Information [file CHEM-31-e02602-s001.pdf]

Supporting information

Coronene Bisimide as a Visible-Light-Mediated Triplet-Triplet Energy Transfer Catalyst

Divya P. Sukumaran, [†] *and Frank Würthner*[†‡] *

[†] Institut für Organische Chemie, Universität Würzburg, Am Hubland, 97074 Würzburg, Germany

[‡] Center for Nanosystems Chemistry (CNC), Universität Würzburg, 97074 Würzburg, Germany

*E-mail: frank.wuerthner@uni-wuerzburg.de

Table of Contents

1. Materials and methods.. 3
2. NMR spectra 5
3. Mechanistic investigation.. 12
4. Optical spectroscopy 14
5. Photosensitization characteristics.. 15
6. References 17

Materials and methods

Chemicals: All chemicals and solvents were purchased from commercial suppliers and were used without any further purification. Maleic anhydride was purified by recrystallization in chloroform and *n*-hexane before irradiation experiments. **CBI-1** was synthesized according to the previously reported procedure.[1] Deuterated solvent used for the reactions was degassed by freeze-pump-thaw and stored in the glovebox.

NMR spectroscopy: NMR spectra were recorded on Bruker Avance III HD 400 MHz spectrometer at 295 K. Chemical shift data are reported in parts per million (ppm, δ scale) downfield from tetramethylsilane and referenced internally to the residual proton (for proton NMR) of the solvent (CDCl3: δ = 7.26).

Purification: Column chromatography was performed with commercial glass columns using silica-gel (particle size 0.040–0.063 mm) with analytical grade solvents as eluents. Gel permeation chromatography was performed with a JAI chromatography system (LC-5060, LaboACE) equipped with three preparative Agilent columns (PL gel 10 µm) in a row using using chloroform (HPLC grade, stabilized with 0.1 % EtOH) as eluent and 6 mL/min flow rate.

UV–vis absorption spectroscopy: UV–vis absorption spectra were recorded under ambient conditions using solvents of spectroscopic grade on JASCO V770 spectrometer with a PAC-743R Peltier for temperature control.

Steady-state fluorescence spectroscopy: Fluorescence spectra were recorded on an Edinburgh Instruments FLS981 fluorescence spectrometer. For long PL lifetimes (>50 ns) a microsecond flash lamp µF2 was used. The fitting was carried out using the tail-fit routine supplied by Edinburgh Instruments Ltd., Inc. For measurements at low temperatures, the Oxford Instruments cryostat Optistat DN with the temperature controller Mercury iTC was used. For the spectra recorded under nitrogen, the solvent was degassed by freeze-pump-thaw and the samples were prepared in the glovebox.

Experimental methods: All the reported photoreactions were carried out under inert conditions (except for oxidation reactions) in pressure NMR tubes and monitored using NMR spectroscopy after irradiation. Oxidation of thioanisole was carried out using oxygen-enriched $CDCl_3$, by bubbling O_2 into the solvent. After preparing the reaction solutions inside glovebox, NMR tubes were irradiated from the bottom using a custom-build light source with high-power LED (LT-1966 OSRAM Oslon SSL 80 green on Star with LT-2020 OSRAM Lisa2 Pin Optic 36°). For every reaction, control experiments without addition of **CBI-1** were carried out under the same concentrations of substrates and irradiation conditions. Products were identified from monitoring the reaction solutions using NMR spectroscopy and the spectral data were matched with previously reported values for corresponding compounds.

Light source characterization: Characterization of LED setup was performed with a PM 200 optical power meter with S121C sensor (THORLABS) and a CCs 200/M wide range spectrometer (THORLABS). A power density of ~113 mW/cm^2 (power 80 mW, area 0.7088 cm^2) was measured at ~2.5 cm from LED and the peak wavelength is 520 nm.

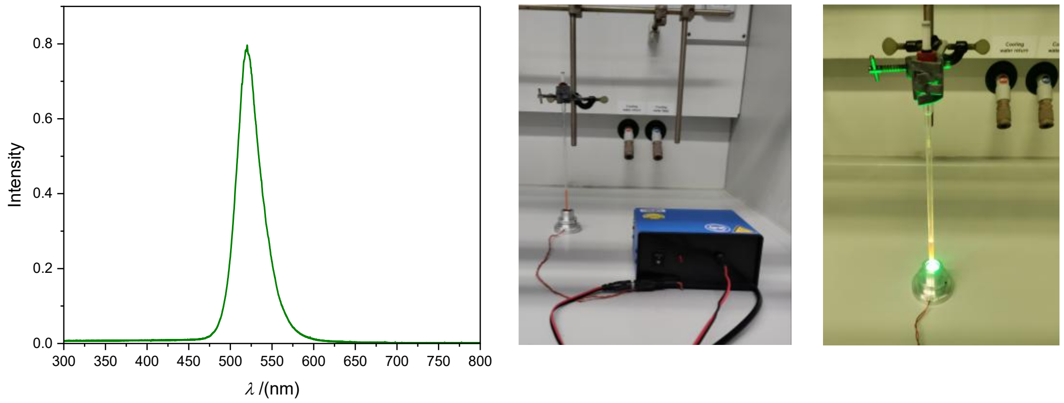

Figure S1. Spectrum of LED irradiation measured along with the LED setup used for irradiation.

Procedure for anthracene dimerization in lab scale:

CBI-1 (0.53 mg, 0.46 µmol, 0.38 mol%) was dissolved in chloroform (40 mL) and degassed using freeze-pump-thaw cycles. Anthracene (21.3 mg, 119 µmol) was added under nitrogen flow and the reaction mixture was degassed again. The reaction mixture was irradiated for 49 hours, and 40 mL hexane was added to the reaction mixture and used directly for purification. The mixture of product and sensitizer was purified using column chromatography with 1:1 mixture of chloroform and *n*-hexane. The fractions were characterized using NMR spectroscopy after purification.
Yield of dianthracene: 81.3 % (17.3 mg, 48.5 µmol); recovery yield of **CBI-1** after purification: 92.4 % (0.49 mg, 0.42 µmol).

Solubility test:

Table S1. Solubility of **CBI-1** at room temperature in some commonly used solvents

Solvent	Solubility	Approximate solubility
Chloroform	soluble	≥5 mg/mL
Dichloromethane	soluble	≥1 mg/mL
Tetrahydrofuran	soluble	~ 0.5 mg/mL
Toluene	very sparingly soluble	<0.05 mg/mL (slight precipitation over time)
Dimethylformamide	very sparingly soluble	< 0.03 mg/mL (slight precipitation over time)
Dimethyl sulfoxide	insoluble	-
Acetonitrile	insoluble	-

NMR spectra

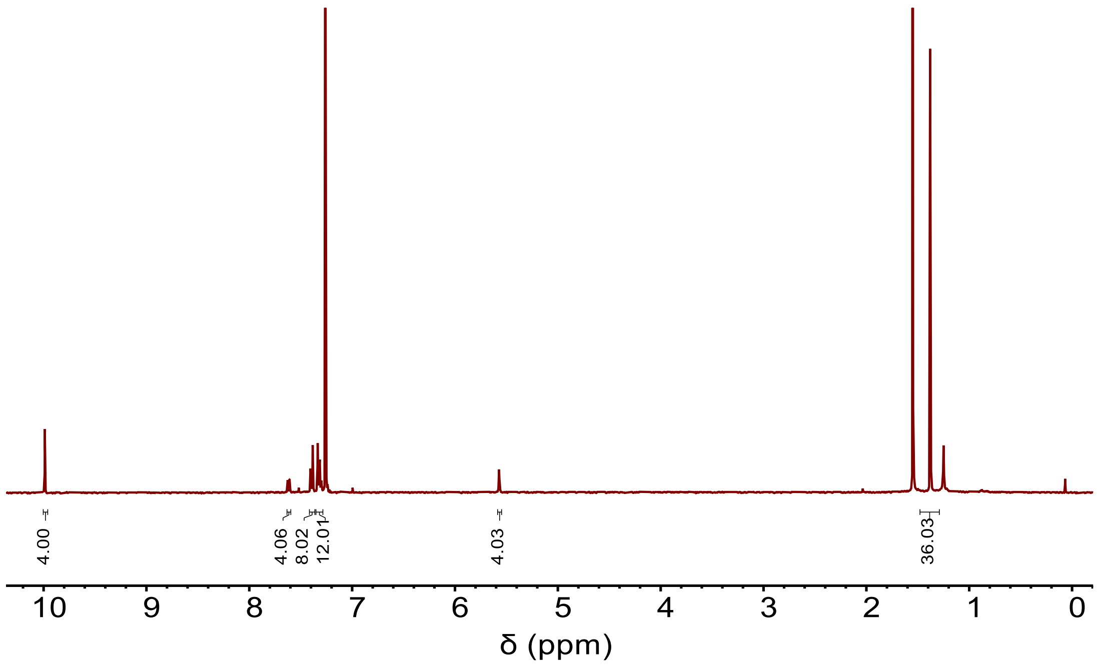

Figure S2. 400 MHz ^{1}H NMR spectrum of the **CBI-1** in $CDCl_3$ at 295 K under ambient conditions.

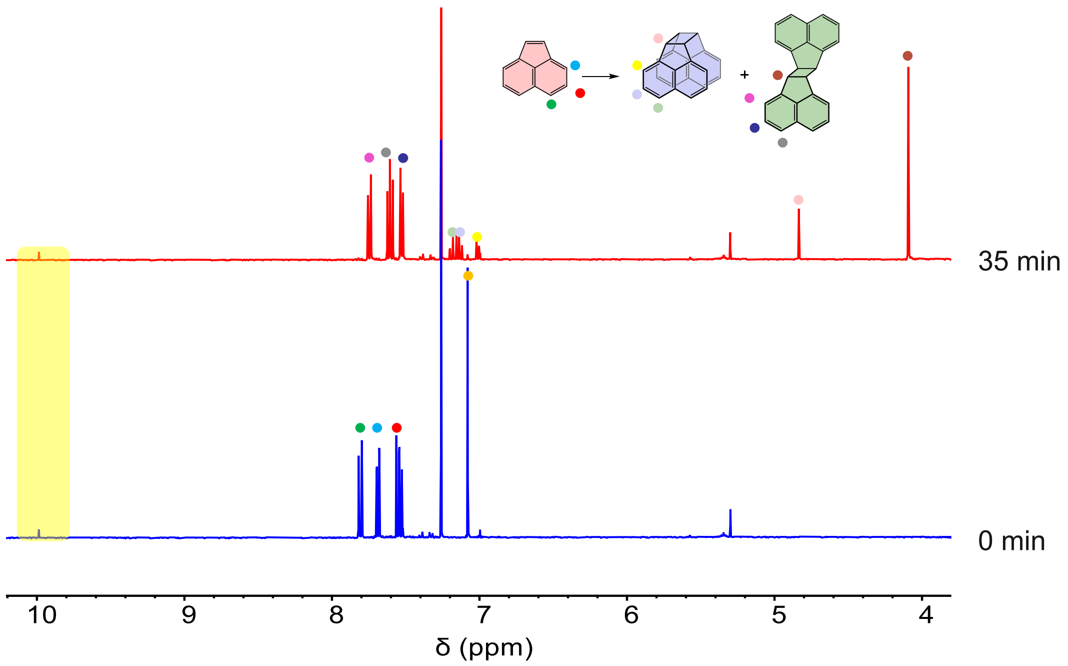

Figure S3. ^{1}H NMR spectra (400 MHz, $CDCl_3$, 295 K, N_2) of acenaphthylene with **CBI-1** ([acenaphthylene] = 2.6×10^{-3} M, [**CBI-1**] = 3.0×10^{-5} M) after irradiating using LED for the mentioned time. Peak corresponding to the protons of coronene core in **CBI-1** is highlighted in yellow.

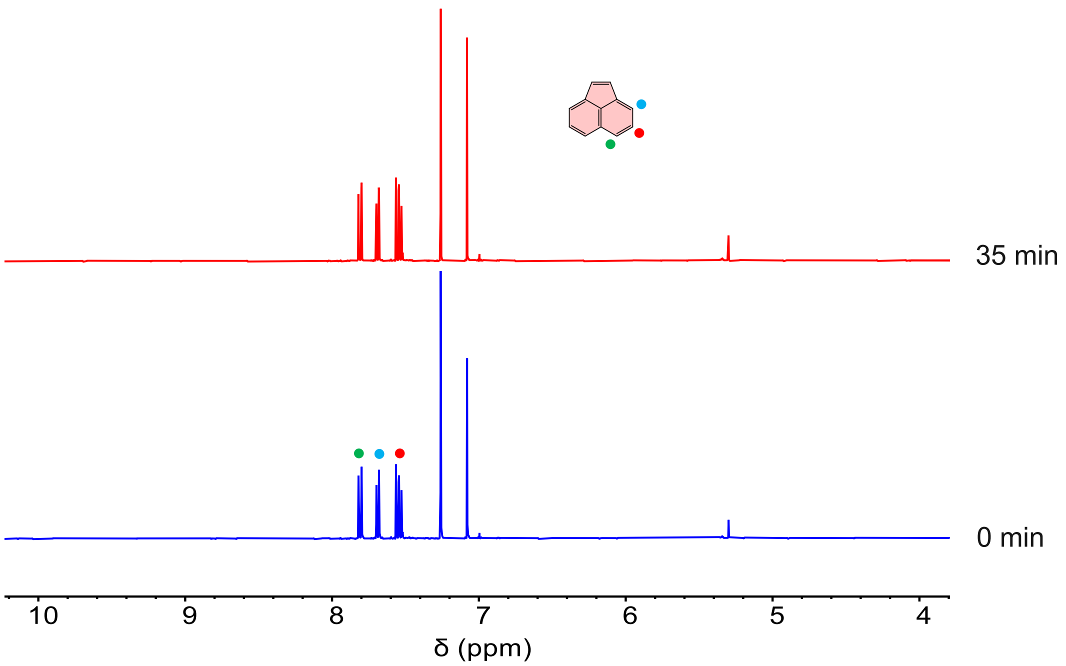

Figure S4. ^{1}H NMR spectra (400 MHz, $CDCl_3$, 295 K, N_2) of acenaphthylene without **CBI-1** ([acenaphthylene] = 2.6×10^{-3} M) after irradiating using LED for the mentioned time.

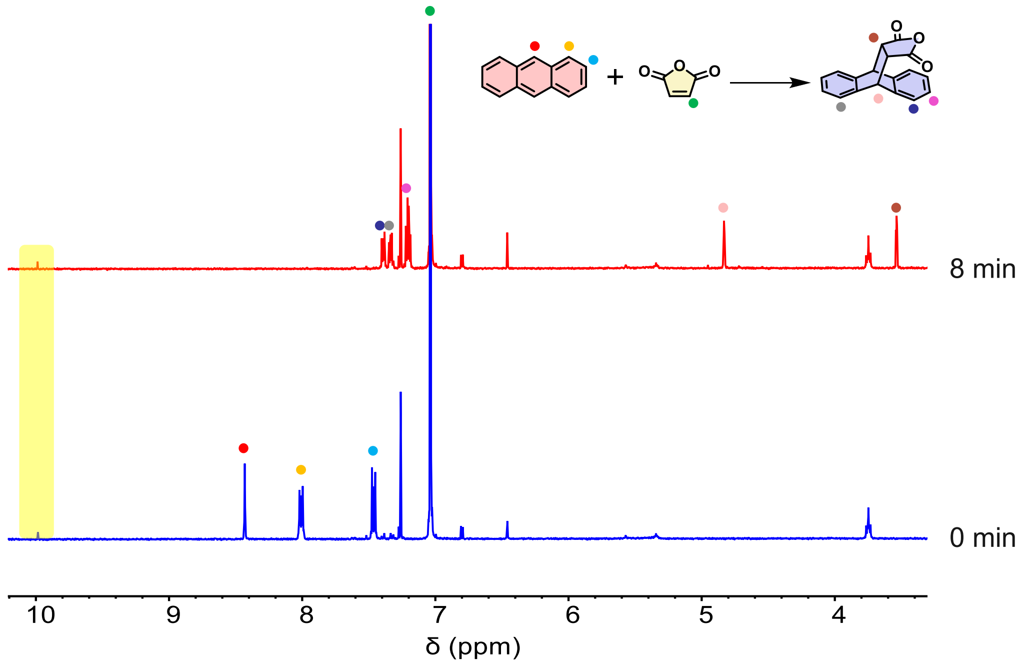

Figure S5. ^{1}H NMR spectra (400 MHz, $CDCl_3$, 295 K, N_2) of anthracene and maleic anhydride with **CBI-1** ([anthracene] = 1.2×10^{-3} M, [maleic anhydride] = 4.0×10^{-2} M, [CBI-1] = 2.8×10^{-5} M) after irradiating using LED for the mentioned time. Peak corresponding to the protons of coronene core in **CBI-1** is highlighted in yellow.

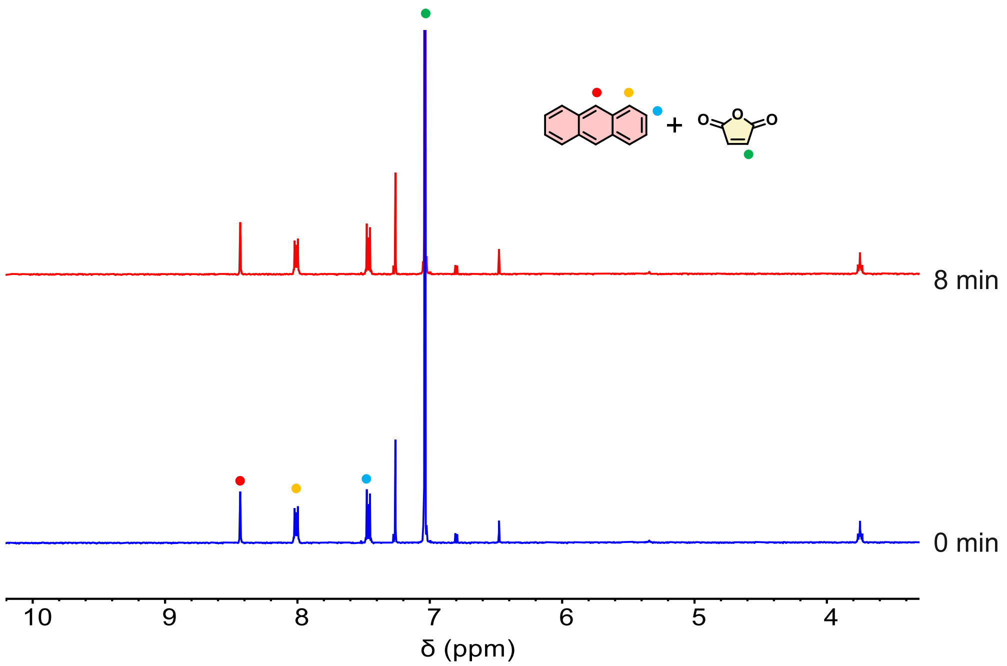

Figure S6. ^{1}H NMR spectra (400 MHz, $CDCl_3$, 295 K, N_2) of anthracene and maleic anhydride without **CBI-1** ([anthracene] = 1.2×10^{-3} M, [maleic anhydride] = 4.0×10^{-2} M) after irradiating using LED for the mentioned time.

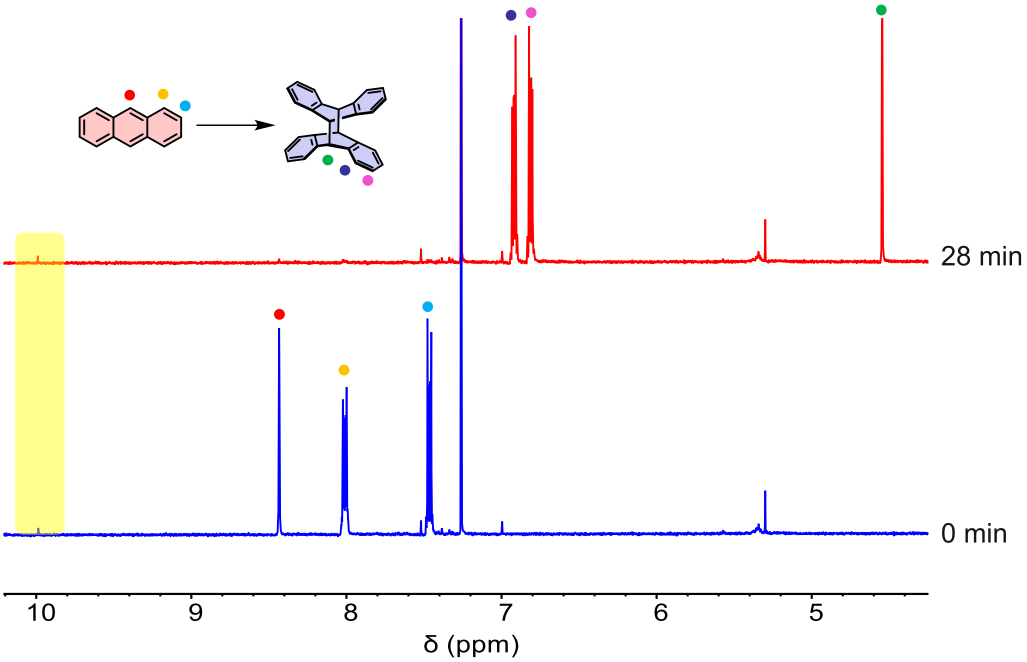

Figure S7. ^{1}H NMR spectra (400 MHz, $CDCl_3$, 295 K, N_2) of anthracene with **CBI-1** ([anthracene] = 1.5×10^{-3} M, [**CBI-1**] = 3.0×10^{-5} M) after irradiating using LED for the mentioned time. Peak corresponding to the protons of coronene core in **CBI-1** is highlighted in yellow.

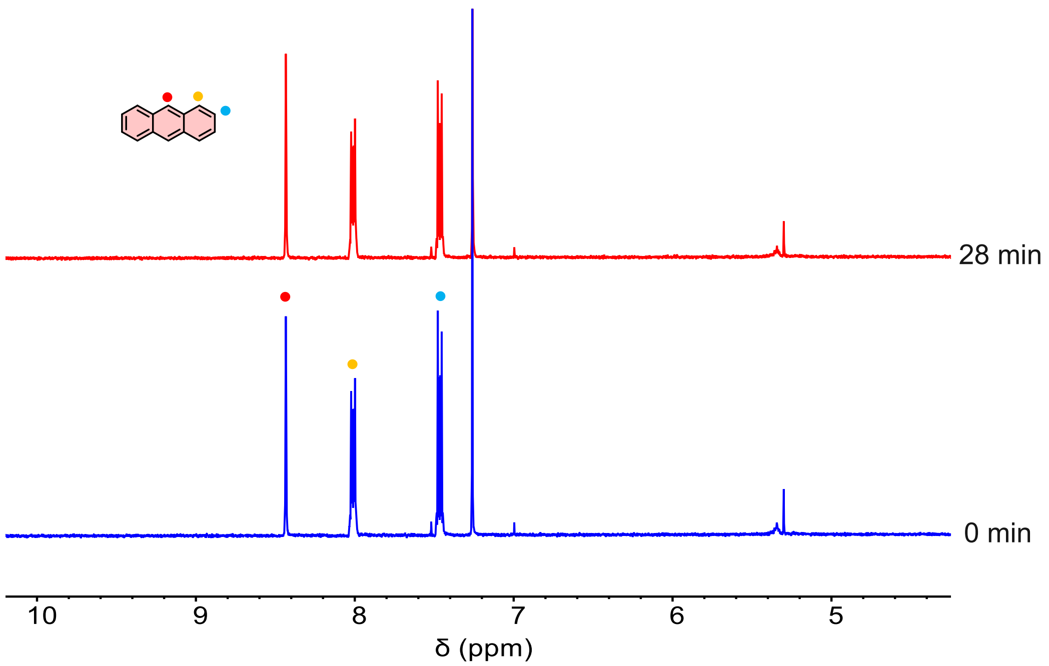

Figure S8. ^{1}H NMR spectra (400 MHz, $CDCl_3$, 295 K, N_2) of anthracene without **CBI-1** ([anthracene] = 1.5 × 10^{-3} M) after irradiating using LED for the mentioned time.

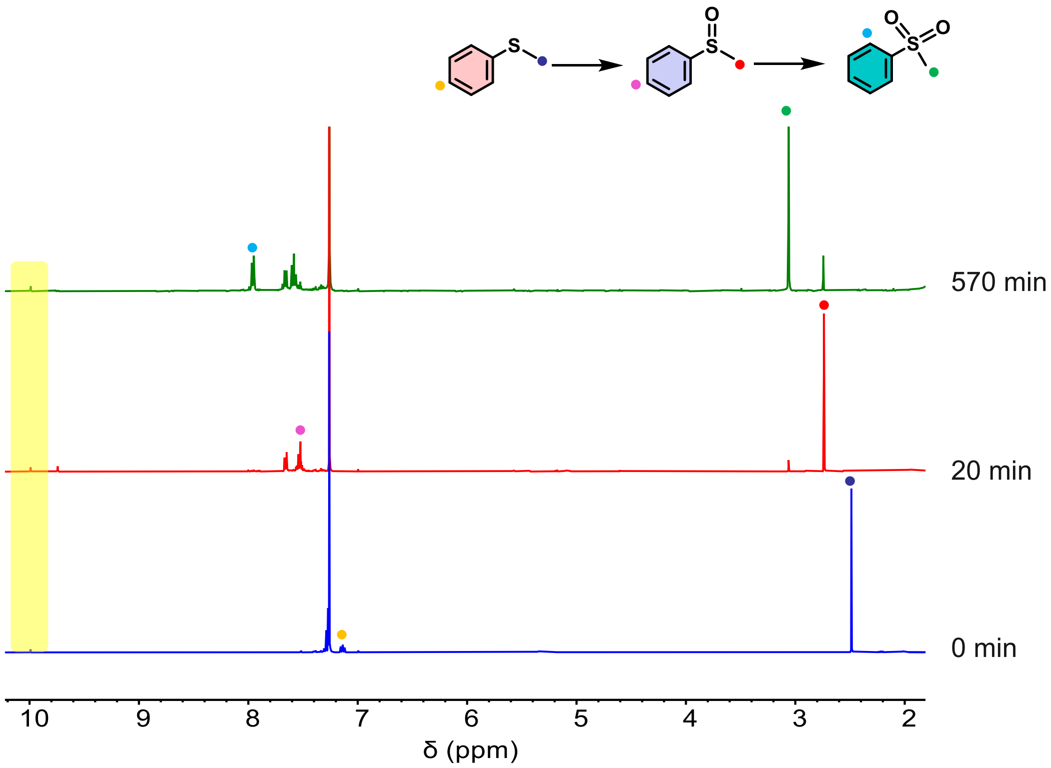

Figure S9. ^{1}H NMR spectra (400 MHz, $CDCl_3$, 295 K, O_2) of thioanisole with **CBI-1** ([thioanisole] = 3.9 × 10^{-3} M, [**CBI-1**] = 3.4 × 10^{-5} M) after irradiating using LED for the mentioned time. Peak corresponding to the protons of coronene core in **CBI-1** is highlighted in yellow.

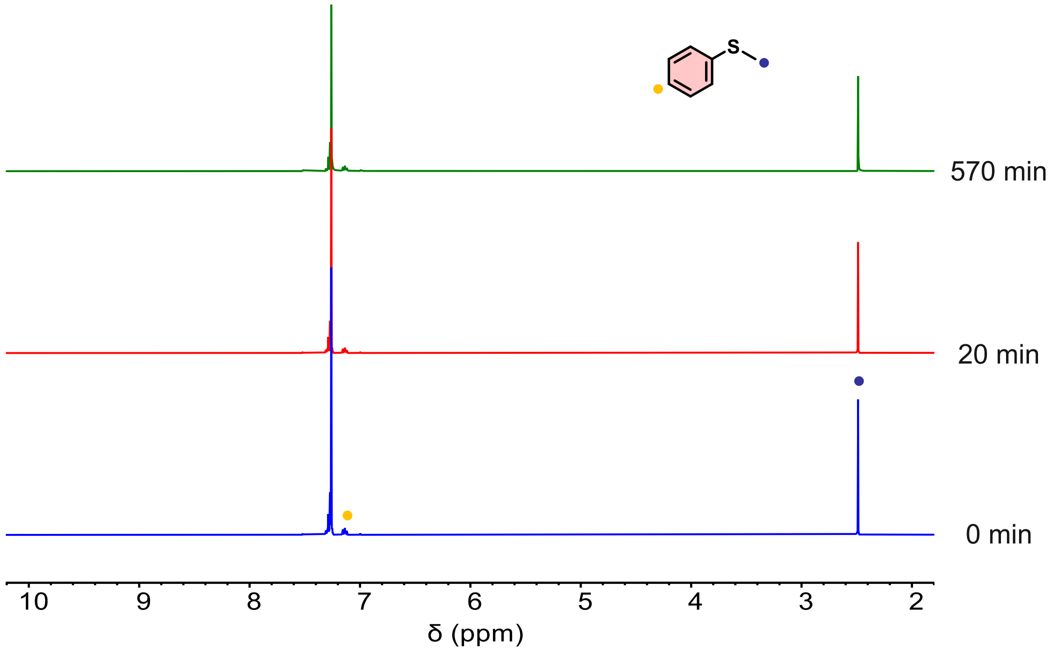

Figure S10. ^{1}H NMR spectra (400 MHz, $CDCl_3$, 295 K, O_2) of thioanisole without **CBI-1** ([thioanisole] = 3.9 × 10^{-3} M) after irradiating using LED for the mentioned time.

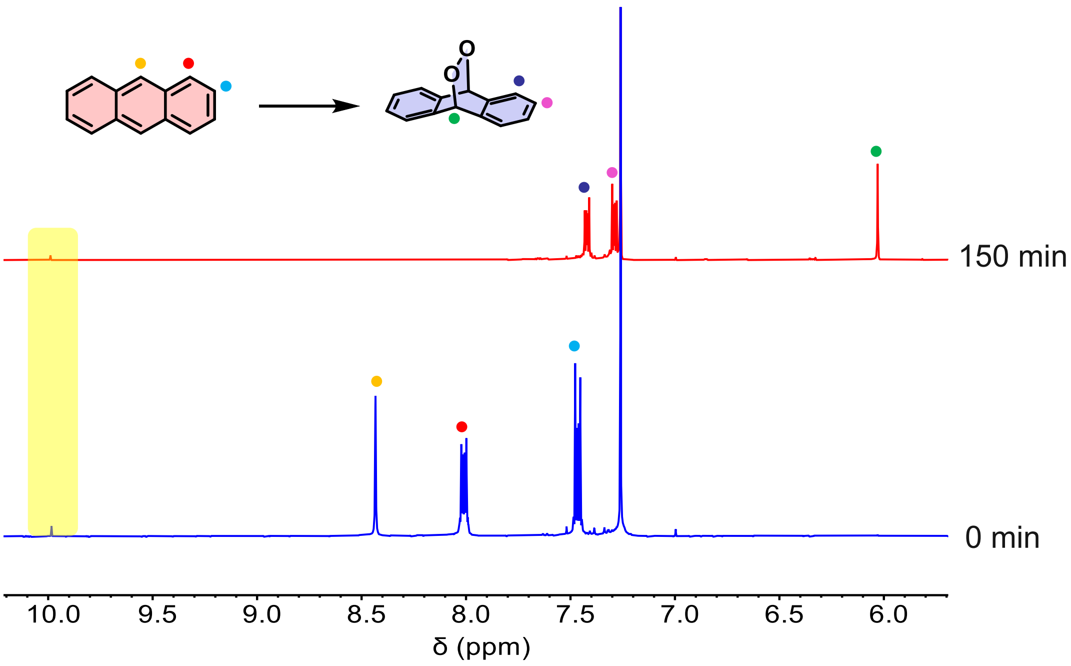

Figure S11. ^{1}H NMR spectra (400 MHz, $CDCl_3$, 295 K) of anthracene with **CBI-1** ([anthracene] = 2.6 × 10^{-3} M, [**CBI-1**] = 3.3 × 10^{-5} M) after ambient light irradiation and atmosphere for the mentioned time. Peak corresponding to protons of coronene core in **CBI-1** is highlighted in yellow.

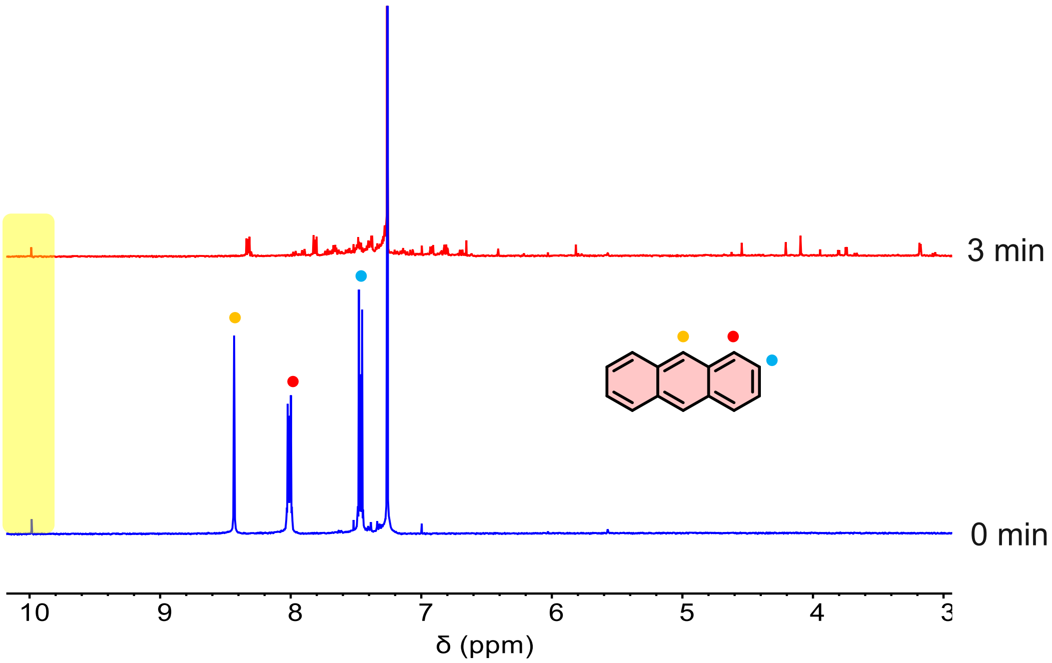

Figure S12. ^{1}H NMR spectra (400 MHz, $CDCl_3$, 295 K) of anthracene with **CBI-1** ([anthracene] = 2.6 × 10^{-3} M, [**CBI-1**] = 3.3 × 10^{-5} M) after irradiating using LED for the mentioned time under ambient conditions. The top spectra show the undesired overoxidized products of anthracene for which peaks were not assigned. Peak corresponding to the protons of coronene core in **CBI-1** is highlighted in yellow.

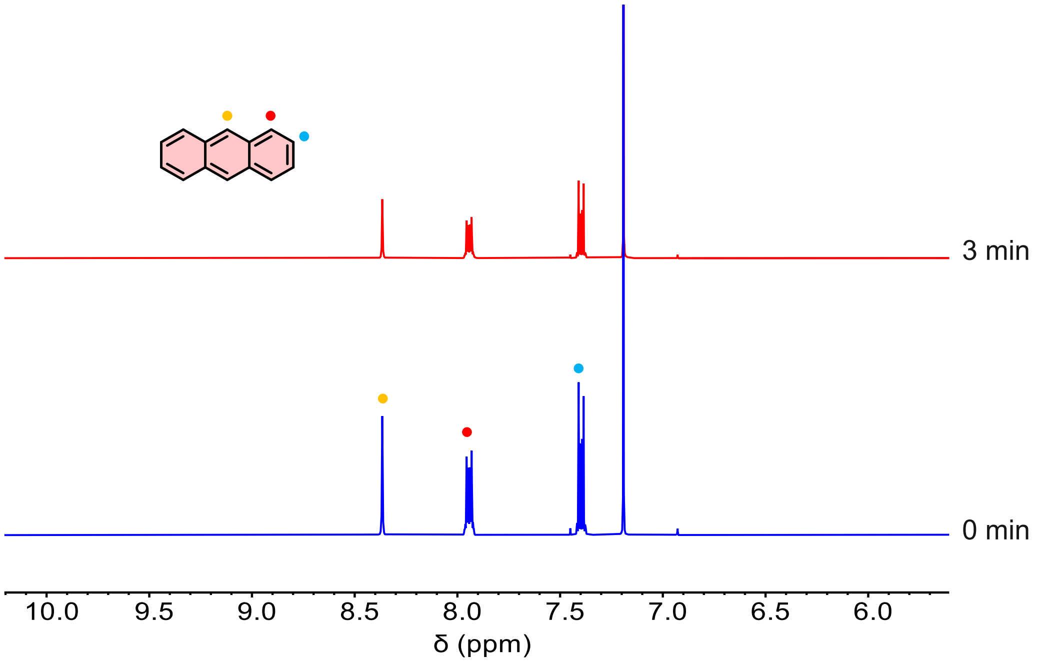

Figure S13. ^{1}H NMR spectra (400 MHz, $CDCl_3$, 295 K) of anthracene without **CBI-1** ([anthracene] = 2.6 × 10^{-3} M) after irradiating using LED for the mentioned time under ambient conditions.

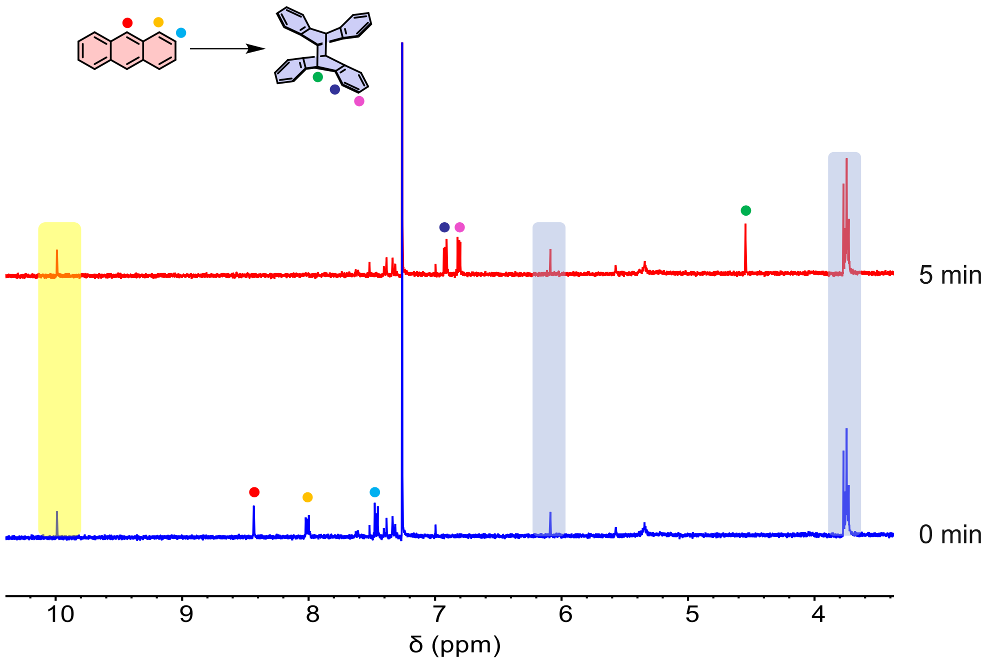

Figure S14. ^{1}H NMR spectra (400 MHz, $CDCl_3$, 295 K, N_2) of anthracene with **CBI-1** and 1,3,5-trimethoxybenzene as internal standard ([anthracene] = 1.3×10^{-4} M, [**CBI-1**] = 2.6×10^{-5} M, [trimethoxybenzene] = 6.9×10^{-5}) after irradiating using LED for the mentioned time. Peak corresponding to the protons of coronene core in **CBI-1** is highlighted in yellow and 1,3,5-trimethoxybenzene is highlighted in blue. Integration of signals referencing internal standard shows that **CBI-1** is not degraded after the reaction.

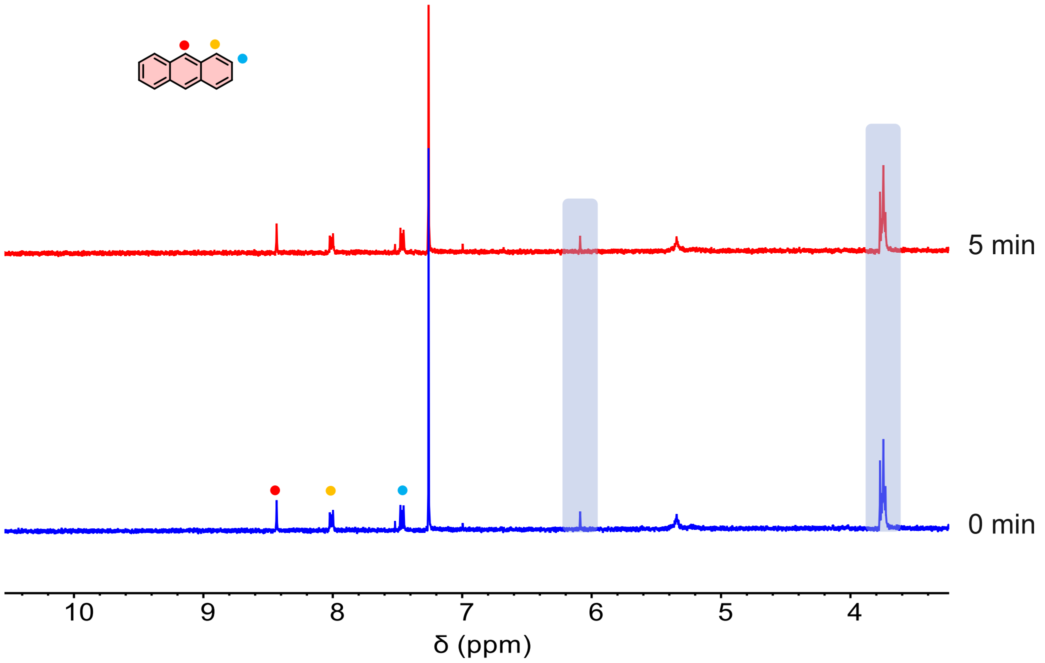

Figure S15. ^{1}H NMR spectra (400 MHz, $CDCl_3$, 295 K, N_2) of anthracene without **CBI-1** and with 1,3,5-trimethoxybenzene as internal standard ([anthracene] = 1.3×10^{-4} M, [trimethoxybenzene] = 6.9×10^{-5}) after irradiating using LED for the mentioned time. Peak corresponding to the protons of 1,3,5-trimethoxybenzene is highlighted in blue.

Mechanistic investigation

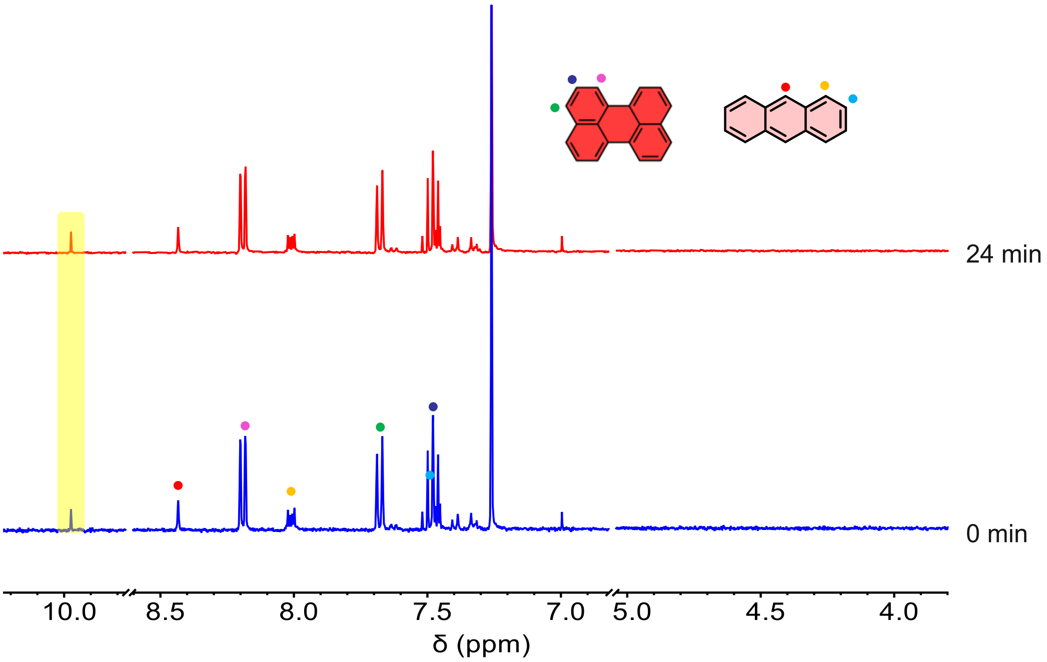

Figure S16. ^{1}H NMR spectra (400 MHz, $CDCl_3$, 295 K, N_2) of anthracene with **CBI-1** and perylene ([anthracene] = 1.6 × 10^{-4} M, [**CBI-1**] = 3.0 × 10^{-5} M, [perylene] = 5.0 × 10^{-4} M) after irradiating using LED for the mentioned time. Peak corresponding to the protons of coronene core in **CBI-1** is highlighted in yellow.

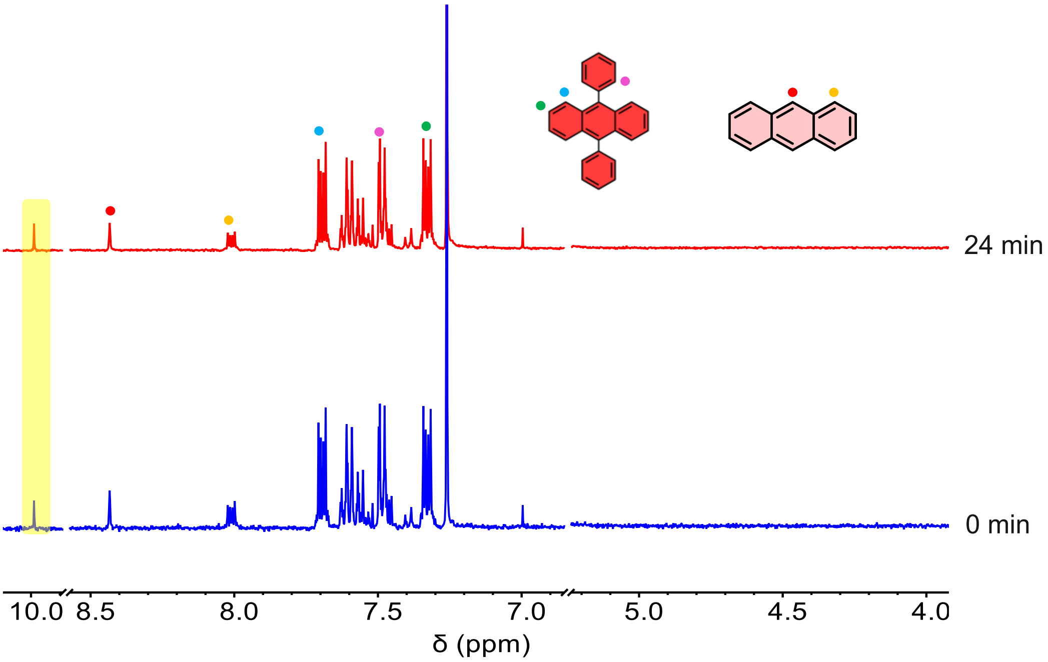

Figure S17. ^{1}H NMR spectra (400 MHz, $CDCl_3$, 295 K, N_2) of anthracene with **CBI-1** and diphenylanthracene ([anthracene] = 1.6 × 10^{-4} M, [**CBI-1**] = 3.0 × 10^{-5} M, [diphenylanthracene] = 5.0 × 10^{-4} M) after irradiating using LED for the mentioned time. Peak corresponding to the protons of coronene core in **CBI-1** is highlighted in yellow.

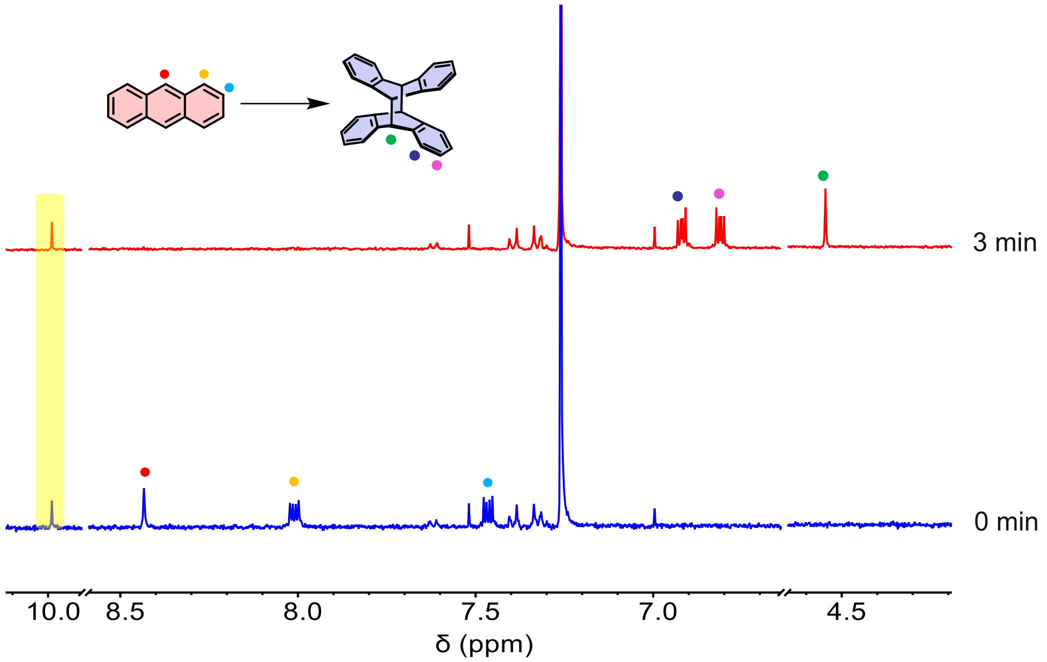

Figure S18. ^{1}H NMR spectra (400 MHz, $CDCl_3$, 295 K, N_2) of anthracene with **CBI-1** ([anthracene] = 1.6×10^{-4} M, [**CBI-1**] = 3.0×10^{-5} M) after irradiating using LED for the mentioned time. Peak corresponding to the protons of coronene core in **CBI-1** is highlighted in yellow.

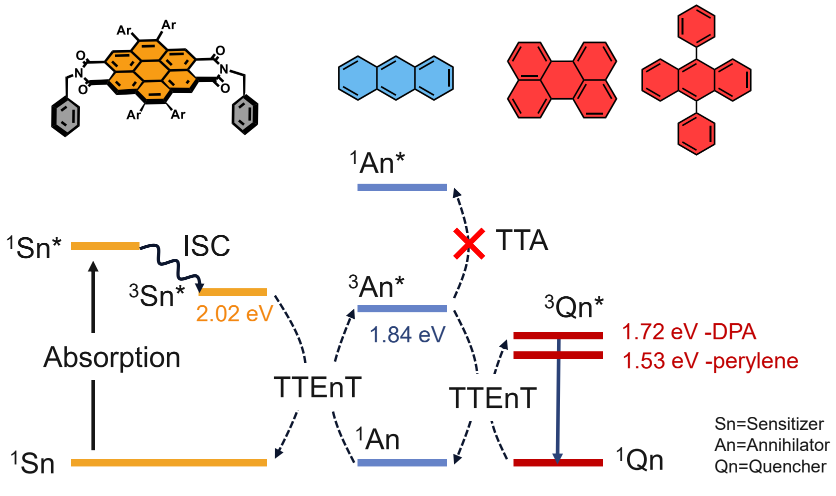

Figure S19. Mechanistic pathway showing the quenching of anthracene triplets by perylene and diphenyl anthracene.

Optical spectroscopy

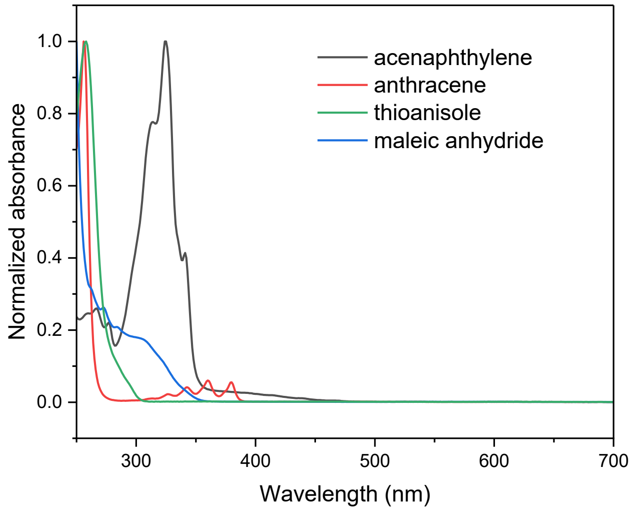

Figure S20. UV–vis absorption spectra of the reactant substrates in chloroform at 295 K.

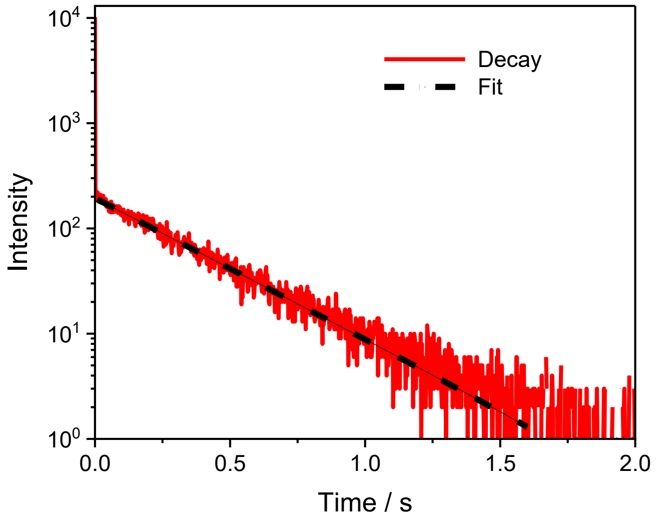

Figure S21. Phosphorescence lifetime decay profile of **CBI-1** in $CHCl_3$ with 20 % ethyl iodide at 77 K (λ_{ex}=530 nm)

Photosensitization characteristics

Table S2. Turnover of reactions using **CBI-1** as photosensitizer

Reaction	[substrate] (10^{-3} M)	[**CBI-1**] (10^{-5} M)	TOF (min^{-1})
Acenaphthylene dimerization	2.6	3.0	2.85
Diels-Alder reaction	1.2	2.8	5.44
Anthracene dimerization	1.5	3.0	1.67
Thioanisole oxidation	1.1	3.5	6.56
Anthracene oxidation	2.6	3.3	0.69

All reactions except oxidations were carried out under inert atmosphere using light irradiation in $CDCl_3$.
Conversion of substrate monitored and calculated using ^{1}H NMR.
Turnover frequency calculated using the slope of the percentage conversion plot of substrate against time of irradiation ($TOF = slope \times \frac{[substrate]}{[catalyst]}$). For plots deviating from linearity, initial rate of conversion was used for TOF calculation.

Table S3. Reported photophysical properties of commonly used photosensitizers and **CBI-1**

Photocatalyst	E_T (eV)	Φ_T	ε ($M^{-1}cm^{-1}$)	λ (nm)	$\tau_{T(rad)}$ *	τ_T
**Heavy-atom-free sensitizers**						
**CBI-1** [1]	2.02	0.39	24,400	529	4.5 ms (RT)<br>(delayed fluorescence)<br>324 ms (77 K, 20 % Et_2I)<br>(phosphorescence)	4.18 μs
Benzophenone [2–5]	2.97	1	19400	335<br>252	1 ms	6.9 μs (n)<br>50 μs (p)
Thioxanthen-9-one [2, 3]	2.74	0.78<br>0.99			8.8 μs	95 μs (n)<br>73 μs (p)
Anthracene [2]	1.84	0.71 (n)<br>0.66 (p)	9700	356	40 ms	670 μs (n)<br>3300 μs (p)
Methylene blue [2, 6]	1.43	0.52	67100	664		450 μs
Xanthone [2]	3.21			370	20 ms	0.02 μs (n)<br>17.9 μs (p)
Acetophenone [2, 4, 5]	3.21	1	12800	241	2.1 ms	0.23 μs
Acridine [2, 4, 5]	1.96 (n)<br>1.94 (p)	0.5 (n)<br>0.82 (p)	11035	356	155 ms (p)	10000 μs (n)<br>14 μs (p)
Benzil [2, 3]	2.31	0.92			5.1 ms (n)<br>5.6 ms (p)	150 μs (n)<br>1500 μs (p)
**Heavy-atom-containing sensitizers**						
$[Ru(bpy)_3]^{2+}$ [7]	2.12	>0.95	14600	452	0.6 μs (RT)	
$Ru(phen)_3{}^{2+}$ [7]	2.18		19000	447	0.9 μs (RT)	
[fac-$Ir(ppy)_3$] [8]	2.5	>0.95	7200	375	1.9 μs (RT)	
$[Ir(bpy)_3](PF6)_3$ [8]	2.4		3300	344	2.4 μs (RT)	
Eosin Y [3–5, 9, 10]	1.96	0.56	112000	527	172 μs (RT)	

* measured at low temperature unless stated otherwise

E_T-triplet energy; Φ_T-ISC yield; $\tau_{T(rad)}$-triplet radiative lifetime (phosphorescence lifetime unless stated); ε-extinction coefficient at mentioned wavelength maxima (λ); τ_T -triplet excited state lifetime

n and p in parenthesis correspond to data in non-polar and polar solvent respectively.

RT-room temperature

References

[1] J. Rühe, K. Vinod, H. Hoh, K. Shoyama, M. Hariharan, F. Würthner, *J. Am. Chem. Soc.* **2024**, *146*, 28222–28232.

[2] M. Montalti, A. Credi, L. Prodi, M. T. Gandolfi, *Handbook of photochemistry*, CRC press, **2006**.

[3] T. Neveselý, M. Wienhold, J. J. Molloy, R. Gilmour, *Chem. Rev.* **2022**, *122*, 2650–2694.

[4] M. Taniguchi, J. S. Lindsey, *Photochem. Photobiol.* **2018**, *94*, 290–327.

[5] M. Taniguchi, H. Du, J. S. Lindsey, *Photochem. Photobiol.* **2018**, *94*, 277–289.

[6] A. Katzenberg, A. Raman, N. L. Schnabel, A. L. Quispe, A. I. Silverman, M. A. Modestino, *React. Chem. Eng.* **2020**, *5*, 377–386.

[7] K. Kalyanasundaram, *Coord. Chem. Rev.* **1982**, *46*, 159–244.

[8] L. Flamigni, A. Barbieri, C. Sabatini, B. Ventura, F. Barigelletti, in *Photochemistry and Photophysics of Coordination Compounds II* (Eds.: V. Balzani, S. Campagna), Springer Berlin Heidelberg, Berlin, Heidelberg, **2007**, pp. 143–203.

[9] S. Dutta, J. E. Erchinger, F. Strieth-Kalthoff, R. Kleinmans, F. Glorius, *Chem. Soc. Rev.* **2024**, *53*, 1068–1089.

[10] A. Penzkofer, A. Beidoun, M. Daiber, *J. Lumin.* **1992**, *51*, 297–314.
